# Exosomal miR‐1246 in Syphilis Serofast State: Diagnostic Value and NLRP3 Inflammasome Suppression

**DOI:** 10.1002/iid3.70434

**Published:** 2026-04-09

**Authors:** Yue Mou, Caifeng He, Fanxiang Wang, Wenhao Cheng, Chaochao Ji, Xinting Wang, Wenlong Hu, Hong Ren

**Affiliations:** ^1^ Department of Dermatology The Affiliated Lianyungang Hospital of Xuzhou Medical University Lianyungang China; ^2^ Department of Dermatology The First Affiliated Hospital (Yijishan Hospital) of Wannan Medical College Wuhu China; ^3^ Department of Dermatology Lianyungang Clinical College of Nanjing Medical University Lianyungang China; ^4^ Department of Dermatology The First Affiliated Hospital of Kangda College of Nanjing Medical University Lianyungang China

**Keywords:** diagnostic value, exosome, miR‐1246, NLRP3 inflammasome, serofast state, syphilis

## Abstract

**Background:**

**S**erofast state (SF), defined as persistent low‐titer antibodies after treatment, poses a diagnostic challenge because of the overlap with serologic features of active infection. Exosomal miRNAs are stable in body fluids and have potential as diagnostic markers.

**Objectives:**

This study aimed to identify plasma exosomal miR‐1246 as a diagnostic biomarker for SF and elucidate its role in NLRP3 inflammasome suppression, providing mechanistic insights into SF pathogenesis.

**Methods:**

Using microarray analysis and reverse transcription quantitative polymerase chain reaction (RT‐qPCR), differential miRNA expression was measured in the plasma samples of SF patients. Microarray analysis, target gene prediction, and Gene Ontology (GO) and Kyoto Encyclopedia of Genes and Genomes (KEGG) pathway analyses were conducted to identify differentially expressed microRNAs (DEmiRNAs). The plasma levels of NLRP3 and related cytokines were quantified using enzyme‐linked immunosorbent assay (ELISA), and the regulatory effect of miR‐1246 on NLRP3 was measured in vitro. Diagnostic performance was assessed based on receiver operating characteristic (ROC) curve analysis for miR‐1246 alone and in combination with the rapid plasma reagin (RPR) test.

**Results:**

Plasma exosomal miR‐1246 was significantly upregulated in SF patients (*p* < 0.001), whereas NLRP3 and its associated factors were downregulated (*p* < 0.05). In vitro experiments confirmed that miR‐1246 negatively regulated NLRP3 inflammasome activity. GO and KEGG analyses revealed that the target genes of DEmiRNAs were involved in multiple biological processes and signalling pathways. ROC analysis showed that miR‐1246 alone yielded an area under the curve (AUC) of 0.760 (sensitivity 77.4%, specificity 62.9%). When combined with RPR, the AUC increased to 0.824 (sensitivity 83.3%, specificity 65.7%).

**Conclusions:**

Exosomal miR‐1246 is elevated in SF and may contribute to its pathogenesis by inhibiting NLRP3 inflammasome. It demonstrates potential as a diagnostic biomarker, particularly when combined with RPR.

## Graphical abstract

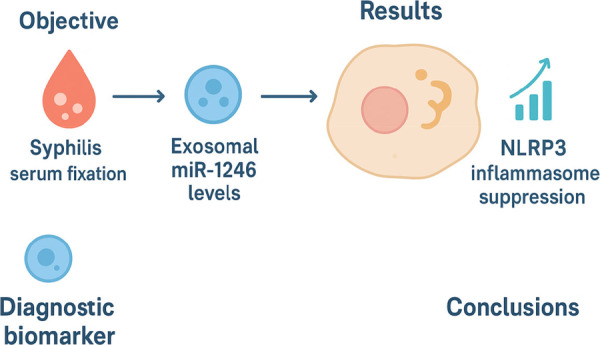

**Abbreviations**
Abbreviation: Fullterm
SF: Serum Fixation
ES: Early Syphilis
SC: Syphilis Cured
HC: Healthy Control
RPR: Rapid Plasma Reagin
TPPA: Treponema pallidum Particle Agglutination
TP: Treponema pallidum
miRNA: microRNA
DEmiRNA: Differentially expressed miRNA
GO: Gene Ontology
KEGG: Kyoto Encyclopedia of Genes and Genomes
NLRP3: NOD‐, LRR‐ and pyrin domain‐containing protein 3
IL‐1β: Interleukin‐1 beta
IL‐18: Interleukin‐18
ELISA: Enzyme‐Linked Immunosorbent Assay
TEM: Transmission Electron Microscopy
NTA: Nanoparticle Tracking Analysis
ROC: Receiver Operating Characteristic
AUC: Area Under the Curve
RT‐qPCR: Reverse Transcription quantitative Polymerase Chain Reaction
PBS: Phosphate‐Buffered Saline
PMA: Phorbol 12‐myristate 13‐acetate
ATP: Adenosine Triphosphate
GAPDH: Glyceraldehyde‐3‐Phosphate Dehydrogenase

## 1 Introduction

Syphilis is a chronic, systemic, and sexually transmitted disease caused by the spirochete Treponema pallidum (TP) [1, 2]. It has shown a resurgence in global incidence in recent years, particularly among young people and high‐risk populations. Based on data from the World Health Organization (WHO), approximately 7.1 million populations aged 15 to 49 were infected with syphilis in 2020 [3]. Syphilis poses a significant risk of cross‐transmission with other sexually transmitted diseases, and individuals infected with the spirochete are at a considerably higher risk of contracting HIV [1, 4, 5]. Early diagnosis and timely treatment are crucial for controlling the transmission of syphilis. However, existing serological tests face limitations in terms of both sensitivity and specificity, particularly in differentiating between patients with active infection and those in the “serofast state” (SF) [6, 7].

The SF refers to a condition in which patients with syphilis retain treponemal and non‐treponemal antibodies at low titers (≤ 1:8) after receiving treatment [8]. This phenomenon occurs in nearly 35% to 44% of patients with syphilis [9]. The absence of overt symptoms in patients with SF makes diagnosis and assessment of treatment efficacy challenging. Therefore, the search for novel biomarkers has become an essential focus in studying the diagnosis of syphilis.

Extracellular vesicles (EVs), particularly the subcategory originating from endosomes (exosomes), are a class of microvesicles with diameters ranging from 30 to 150 nanometers. Enclosed within a lipid bilayer, these particles carry biomolecules, such as microRNAs (miRNAs), proteins, and lipids [10, 11, 12]. These vesicles are stable in bodily fluids, which makes them an ideal and non‐invasive method for liquid biopsy [13, 14]. miRNAs, are non‐coding small RNAs of about 22 nucleotides in length that regulate gene expression by binding to specific sequences of target messenger RNAs (mRNAs) [15, 16, 17]. Exosomal miRNAs have demonstrated significant diagnostic and therapeutic potential in the context of cancer, immune diseases, and infectious diseases [18, 19, 20, 21, 22]. Studies have shown that changes in miRNA expression occur during syphilis infection, which may reflect the pathological processes at different stages of syphilis [23, 24, 25].

Our microarray screening indicated that miR‐1246 is significantly upregulated in the plasma exosomes of patients with syphilis SF, suggesting its potential as a biomarker for SF. It was shown that miR‐1246 is a conserved exosome‐associated microRNA that plays a crucial role in regulating immune and inflammatory responses in various pathological processes, including cancer, autoimmune diseases, and infections [26, 27, 28, 29]. Given its established function in modulating inflammatory signaling, we hypothesized that miR‐1246 may contribute to the pathogenesis of syphilitic SF disease by negatively regulating central inflammatory hubs.

Based on the hypothesis that miR‐1246 may target central inflammatory hubs, we focused on the inflammasome protein 3 (NLRP3), which comprises NOD, LRR, and pyrin domains. As a key cytoplasmic multiprotein complex, the NLRP3 inflammasome plays a critical role in several infectious and chronic inflammatory diseases [26, 30, 31, 32]. Existing studies have shown a strong association between the NLRP3 inflammasome and syphilis infection, suggesting that it may be involved in the pathogenesis of the serofast state [33, 34, 35, 36].

In conclusion, this study aimed to explore the molecular mechanisms by which miR‐1246 may regulate the NLRP3 inflammasome during the development of syphilis SF and assess its diagnostic value in SF. This study provides new insights into the early diagnosis and treatment of syphilis.

## 2 Materials and Methods

### 2.1 Patients and Plasma Samples

From June 2020 to September 2024, we collected blood samples from 201 patients with syphilis in the dermatology department of Lianyungang Hospital, affiliated with Xuzhou Medical University, and Yijishan Hospital of Wannan Medical College. The patients were divided into three groups based on the diagnostic criteria for syphilis of the National Health Commission of China (2018) [37] as follows: 79 patients with early syphilis (ES), 93 patients with SF, and 29 patients with syphilis cured (SC). The ES group included patients who were positive for both RPR and TPPA, with or without clinical symptoms, such as primary chancre or skin lesions. The SF group consisted of patients with low titers (≤ 1:8) of treponemal and non‐treponemal antibodies after standard treatment. The SC group included patients whose treponemal and non‐treponemal antibodies became negative after treatment. During the same period, 38 healthy volunteers were enrolled in the healthy control (HC) group. Pregnant women, lactating women, patients with HIV/AIDS, autoimmune diseases, cancer, or systemic infections were excluded [20]. All samples (5 mL of peripheral blood) were collected using EDTA anticoagulant tubes and processed within 1 h after collection. Plasma samples were separated via centrifugation at 3000×g for 10 min and stored at −80°C. This study was approved by the ethics committees of Lianyungang Hospital Affiliated with Xuzhou Medical University and Yijishan Hospital of Wannan Medical College, and all participants provided written informed consent.

### 2.2 Isolation of Plasma Exosomes

Using the Total Exosome Isolation Reagent (from plasma) (Invitrogen), exosomes were isolated from 200 µL of plasma following the manufacturer's instructions. The specific procedure was as follows: The plasma sample was centrifuged at 2000×g for 20 min to remove cellular debris. The supernatant was removed and centrifuged again at 10000 × g for 20 min. The resulting clear supernatant was transferred to a fresh tube. It was thoroughly mixed with 0.5 volumes of phosphate‐buffered saline (PBS). Then, 0.2 volume of exosome precipitation reagent was added to the sample (the total volume was equal to the sum of plasma and PBS). The sample was vortexed to ensure complete mixing. After incubation at room temperature for 10 min, the sample was centrifuged at 10,000×g at room temperature for 5 min. The pellet was resuspended in 100 μL of PBS and stored at −80°C for subsequent analysis.

### 2.3 Transmission Electron Microscopy (TEM)

A 20 uL aliquot of the resuspended exosome sample was placed on a carbon‐coated copper grid. Excess liquid was removed after settling for 1 min. The sample was then fixed with 1% glutaraldehyde for 5 min, washed three times with water, and stained with 3% phosphotungstic acid for 3 min, followed by three more washes with water. After drying at room temperature, the samples were examined by TEM (Thermo Fisher).

### 2.4 Nanoparticle Tracking Analysis (NTA)

The sample chamber was cleaned using particle‐free Duchenne phosphate buffer solution before injecting the diluted exosome sample. Exosome size and concentration were measured at a pressure of 700 Pa and calibrated using the CPC100. Particle size distribution was analyzed using the NanoSight N300 (Malvern, UK).

### 2.5 Extraction of miRNA From Plasma Exosomes

Exosomal RNA was extracted using the exoRNeasy Midi Kit (QIAGEN). Equal volumes of plasma and XBP were mixed and centrifuged to remove the supernatant. The remaining pellet was washed with 3.5 mL XWP. The lysate was collected after further washing with QIAzol, mixed with chloroform, and centrifuged at 4°C for 15 min. The upper aqueous phase was collected, mixed with ethanol, and transferred to a new RNeasy MinElute column. The RNA sample was eluted with 20 µL of RNase‐free water. The concentration of total RNA, including miRNA, was quantified using a spectrophotometer (Thermo Fisher).

### 2.6 miRNA Microarray Analysis

miRNA microarray analysis was conducted by Shanghai Bohao Biotechnology Co., LTD (Shanghai, China) using an Agilent 2100 Bioanalyzer instrument (Agilent Technologies Inc.). DEmiRNAs were conducted based on the criteria of a fold change and a *p*‐value calculated using a t‐test. *p* < 0.05 and |logFC | ≥ 1 were regarded as the threshold.

### 2.7 miRNA Target Gene and Pathway Enrichment Analysis

The miRWalk tool was employed to conduct a predictive analysis of potential target genes for microRNAs. Subsequently, the target genes were subjected to GO and KEGG pathway enrichment analysis via the DAVID database. Concurrently, visualization was achieved through the MicroBioinformatics platform to reveal their biological functions and potential pathways.

### 2.8 Reverse Transcription Quantitative Polymerase Chain Reaction (RT‐qPCR)

For total RNA extraction from plasma exosomes, refer to the section ‘Extraction of miRNA from plasma exosomes’. Total RNA from THP‐1‐derived macrophages was extracted using Trizol reagent (Invitrogen) following the manufacturer's instructions. RNA concentration and purity were determined using a spectrophotometer (ThermoFisher). For reverse transcription and quantitative PCR, RNA was transcribed into cDNA using the PrimeScript™ RT Kit (Takara) and miRNA‐specific stem‐loop primers. Quantitative PCR was conducted using TB Green® Premix Ex Taq™ II (Takara) on a real‐time fluorescent quantitative PCR system. Reaction conditions were as follows: initial denaturation at 95°C for 30 s, followed by 40 cycles (95°C for 5 s, 60°C for 34 s). For exosome‐derived miRNAs, Cel‐miR‐39 (Proteinbio) served as an exogenous control at a final concentration of 250 nM. For cellular miRNAs, U6 was used as the endogenous control. The relative expression levels of target miRNAs (e.g., miR‐1246) were calculated using the 2^‐ΔΔCT method. Primers used for reverse transcription and qPCR are listed in Supplementary Table S1.

### 2.9 Western Blotting

Protein concentration was measured using the BCA method (Beyotime). After adjustment for protein concentration, protein samples were extracted using RIPA buffer containing protease inhibitors (Beyotime). In total, 20 μg of the protein sample was loaded onto a 10% SDS‐PAGE gel for separation and then transferred to a PVDF membrane. The membrane was blocked with 5% skim milk for 2 h and then incubated with primary antibodies at 4°C overnight. Proteins were detected using ECL (Beyotime) and quantified using ImageJ software after incubation with HRP‐conjugated secondary antibodies (1:5,000, Absin, ABS20040) for 1 h. During exosome characterization, antibodies against positive exosome markers (CD63 [1:1000, Abcam, ab134045], ALIX [1:1000, Abcam, ab275377], and CD81 [1:1000, Abcam, ab109201]) and antibodies against the negative exosome marker calnexin (1:1000, FineTest, FNab01210) were used for detection. To conduct cellular protein analysis, antibodies targeting NLRP3 (1:1000, CST, #15101 T), Caspase‐1 (1:1000, Abcam, ab207802), and GAPDH (1:1000, CST, #5174 T) were utilized to analyze protein lysates from THP‐1‐derived macrophages, with GAPDH serving as the loading control.

### 2.10 Enzyme‐Linked Immunosorbent Assay (ELISA)

The plasma levels of NLRP3, IL‐1β, and IL‐18 were quantified using an ELISA kit (Mlbio) following the manufacturer's instructions. Absorbance values were read at 450 nm.

### 2.11 Culture and Differentiation of THP‐1 Cells

THP‐1 monocytes (laboratory stock) were cultured in RPMI‐1640 medium supplemented with 10% fetal bovine serum and 1% antibiotics at 37°C in an incubator containing 5% CO2. M0 differentiation of macrophages was induced by exposure to 50 ng/mL PMA (Biosharp) for 48 h. After 24 h, the medium was replaced with fresh medium.

### 2.12 Macrophage Transfection and Inflammasome Activation

miR‐1246 mimics and inhibitor (Proteinbio) were transfected into M0 macrophages using Lipofectamine 2000 (Invitrogen). All culture media were replaced 6 h after transfection. Macrophages were stimulated with 10 ng/mL TNF‐α (Peprotech) for 12 h and exposed to 5 mM ATP (MedChemExpress) for 1 h to activate the NLRP3 inflammasome. The sequences of the microRNA mimics and inhibitors used in this study are provided in Supplementary Table S2.

### 2.13 Statistical Analysis

Statistical analysis was conducted using SPSS 25.0 and GraphPad Prism 10.0 software. Measurement data were initially subjected to the Shapiro‐Wilk normality test and the Levene's test for homogeneity of variance. Data sets with a normal distribution were analyzed using one‐way ANOVA, while the Kruskal‐Wallis test was used to analyze data without a normal distribution. Count data were analyzed using the Chi‐squared test. The diagnostic efficacy of miRNA was evaluated using the ROC curve, with *p* < 0.05 indicating a significant difference.

## 3 Results

**Figure 1:**
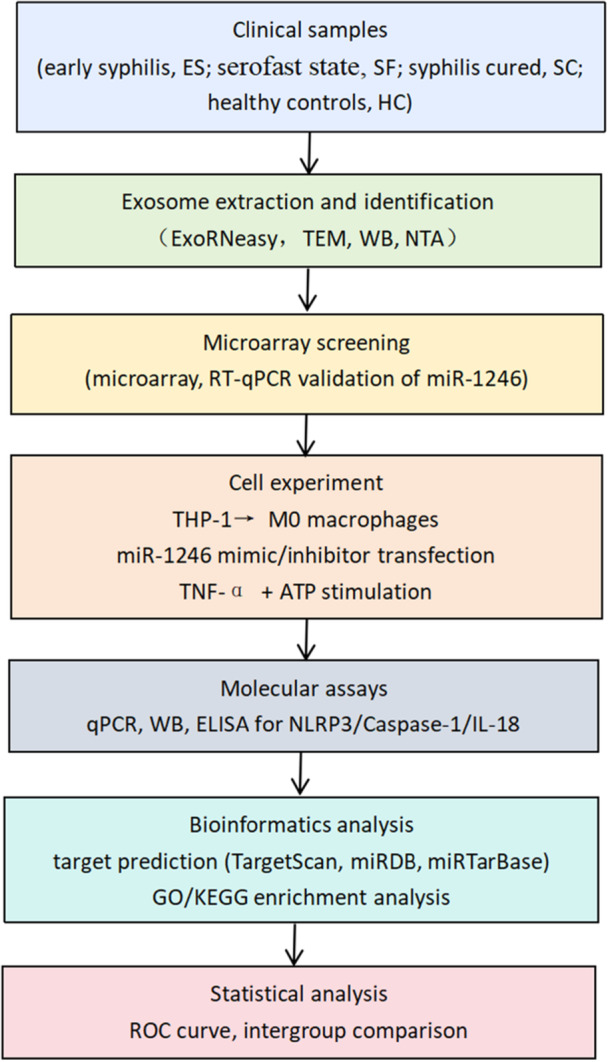
Study design and methodology flowchart.

The methodological flowchart for this study is presented in Figure 1.

### 3.1 Clinical Characteristics of the Subjects

This study comprised 27 subjects who were included in the microarray analysis and 212 subjects who were used for RT‐qPCR validation. The clinical characteristics of all subjects are shown in Table 1. Statistical analysis revealed significant differences in RPR titers between early syphilis (ES) and serum fixation (SF) groups (*p* < 0.001). There were no significant differences between ES, SF, syphilis cured (SC), and healthy control (HC) groups in terms of age or gender (*p* > 0.05).

**Table 1:** Information on the Clinical Samples Used for the Microarray Study and RT‐qPCR Validation Study. (*Data are expressed as mean age ± standard deviation (SD). Gender and RPR titer groups are presented as counts (percentages). *p*‐values for continuous variables (age) were determined using one‐way analysis of variance (ANOVA). Categorical variables (gender, RPR titer distribution) were compared using nonparametric tests (Chi‐square test or rank‐sum test). *p*‐values < 0.05 were considered statistically significant.*).

Microarray study					RT‐qPCR Validation study				
Clinical features	ES (*n* = 9)	SF (*n* = 9)	HC (*n* = 9)	*p*	ES (*n* = 70)	SF (*n* = 84)	SC (*n* = 29)	HC (*n* = 29)	*p*
**Age (years, mean ± SD)**	39.89 ± 12.21	46.11 ± 17.60	42.22 ± 12.55	0.654	36.56 ± 17.04	38.75 ± 12.41	32.45 ± 10.34	37.83 ± 10.02	0.067
**Sex (*n*, %)**				0.017					0.911
Male	3 (33.3)	3 (33.3)	4 (44.4)		36 (51.4)	39 (46.4)	15 (51.7)	15 (51.7)	
Female	6 (66.7)	6 (66.7)	5 (55.6)		34 (48.6)	45 (53.6)	14 (48.3)	14 (48.3)	
**RPR titer (positive/negative, *n*)**	9/0	9/0	0/9	< 0.001	70/0	84/0	0/29	0/29	< 0.001
RPR titer1:128	1	0	0		1	0	0	0	
RPR titer1:64	1	0	0		5	0	0	0	
RPR titer1:32	5	0	0		5	1	0	0	
RPR titer1:16	0	0	0		8	2	0	0	
RPR titer1:8	1	1	0		16	5	0	0	
RPR titer1:4	1	3	0		8	18	0	0	
RPR titer1:2	0	4	0		10	23	0	0	
RPR titer1:1	0	1	0		17	35	0	0	
**ES versus SF RPR titer (*n*, %)**				0.003					< 0.001
RPR titer ≥ 1:8	8 (88.9)	1 (11.1)	—		35 (50.0)	8 (9.5)	—	—	
RPR titer < 1:8	1 (11.1)	8 (88.9)	—		35 (50.0)	76 (90.5)	—	—	

### 3.2 The Results of Exosome Characterization

**Figure 2:**
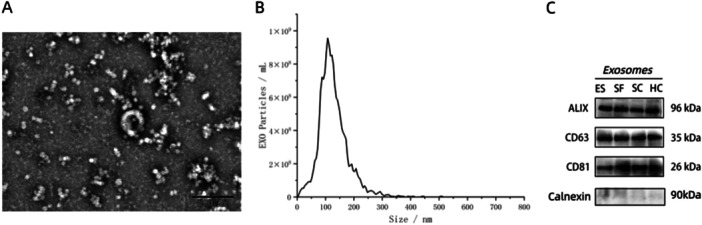
Characterization of EVs. (A) TEM showing the typical morphology and size of EVs isolated from plasma. (B) NTA demonstrates the size distribution and concentration of EVs isolated from plasma. (C) The procedure of Western blotting was implemented to detect the positive EVs markers CD63, CD81, and ALIX, as well as the negative EVs marker Calnexin.

Exosomes were extracted from human plasma using Total Exosome Isolation Reagent (Invitrogen). TEM showed that the exosomes had a round or bowl‐shaped vesicle structure with an intact membrane (Figure 2A). NTA revealed that the exosomes had diameters ranging from 30 to 150 nm (Figure 2B). Western blotting also confirmed the presence of exosomes, evidenced by the presence of positive exosome markers CD63, CD81, and ALIX and the absence of the negative exosome marker calnexin (an endoplasmic reticulum protein) (Figure 2C). Together, these results validated the isolation of exosomes.

### 3.3 The Results of miRNA Microarray Analysis

**Figure 3:**
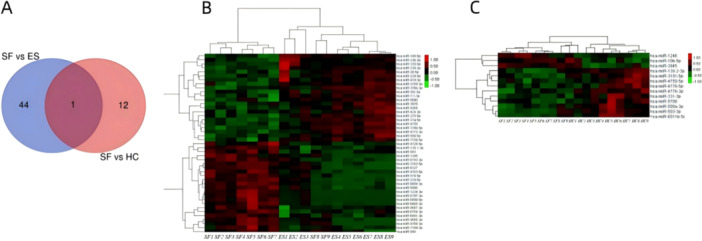
Results of microarray analysis. (A) Venn diagram showing miRNAs with significant differences. (B) Heatmap analysis of differentially expressed miRNAs in SF compared to ES. (C) Heatmap analysis of differentially expressed miRNAs in SF compared to HC.

We performed microarray analysis on samples from nine patients with early syphilis (ES), nine serofixed (SF) patients, and nine healthy control (HC) patients. Venn diagram results showed the presence of a significantly higher number of DEmiRNAs in the SF group compared to both the ES and HC groups (Figure 3A). Heatmap analysis revealed that SF had 45 DEmiRNAs compared to ES (*p* < 0.01, Figure 3B). Compared to HC, SF had 13 DEmiRNAs (*p* < 0.01, Figure 3C). miR‐1246 was significantly upregulated in the SF group and was regarded as a potential exosomal miRNA biomarker for SF.

### 3.4 GO and KEGG Analysis of DEmiRNA Target Genes

**Figure 4:**
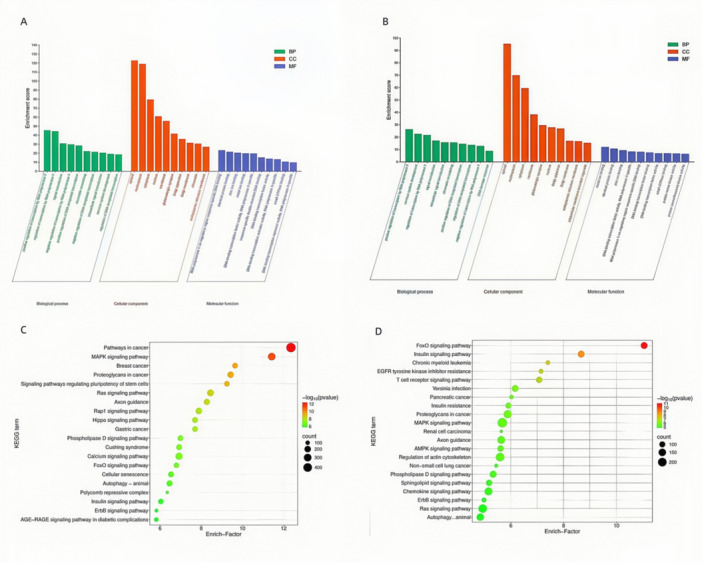
GO and KEGG enrichment analysis of DEmiRNA target genes. (A)GO enrichment of target genes from DEmiRNAs identified in SF versus ES. (B) GO enrichment of target genes from DEmiRNAs identified in SF versus HC. (C) KEGG pathway enrichment bubble plot of target genes from DEmiRNAs in SF versus ES. (D) KEGG pathway enrichment bubble plot of target genes from DEmiRNAs in SF versus HC.

To investigate the potential biological functions of DEmiRNAs, we conducted GO and KEGG enrichment analyses on their predicted target genes using the DAVID database. GO analysis indicated that these biological processes were primarily linked to cellular homeostasis, immune regulation, and stress responses (Figures 4A and 4B). The outcomes of the KEGG pathway analysis are depicted in Figures 4C and 4D. Notably, these comparative studies (SF vs. ES group and SF vs. HC group) indicated the significant enrichment of immune and inflammation‐related pathways, such as the MAPK signaling pathway, Ras signaling pathway, chemokine signaling pathway, and T cell receptor signaling pathway. These pathways are closely associated with innate immune regulation and inflammasome activation. Therefore, the present study supports the hypothesis that extracellular miR‐1246 can exert its biological effects by targeting NLRP3 and modulating inflammasome‐related signaling pathways.

### 3.5 The Expression of miR‐1246 in Plasma Exosomes

**Figure 5:**
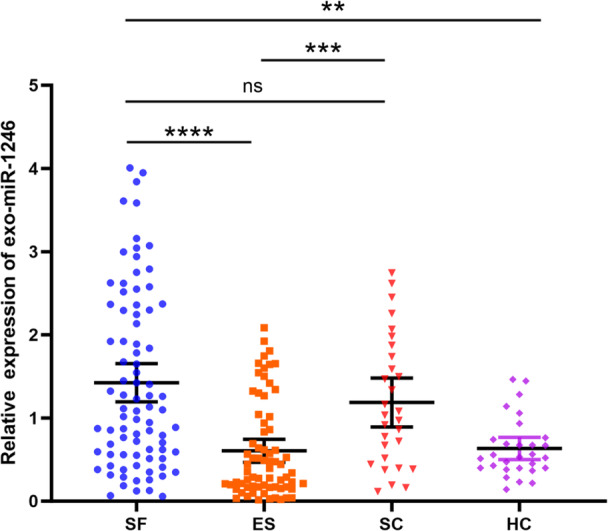
Detection of exosomal miRNAs in different groups by RT‐qPCR. exosomal miR‐1246 expression was measured in 212 samples with cel‐miR‐39 set as an exogenous control. The four groups were statistically analyzed using the Kruskal Wallis test. *p < 0.05.

RT‐qPCR was used to measure the expression levels of miR‐1246 in plasma exosomes (Figure 5). miR‐1246 expression was significantly upregulated in the SF group compared to the ES group (*p* < 0.001) and the HC group (*p* < 0.01). No significant difference was observed between the SF group and the SC group (*p* > 0.05). These results are consistent with the findings of microarray analysis. In addition, miR‐1246 levels were significantly increased in exosomes from the SC group compared to those from the ES group (*p* < 0.001).

### 3.6 The Regulatory Effect of miR‐1246 on the NLRP3 Inflammasome

**Figure 6:**
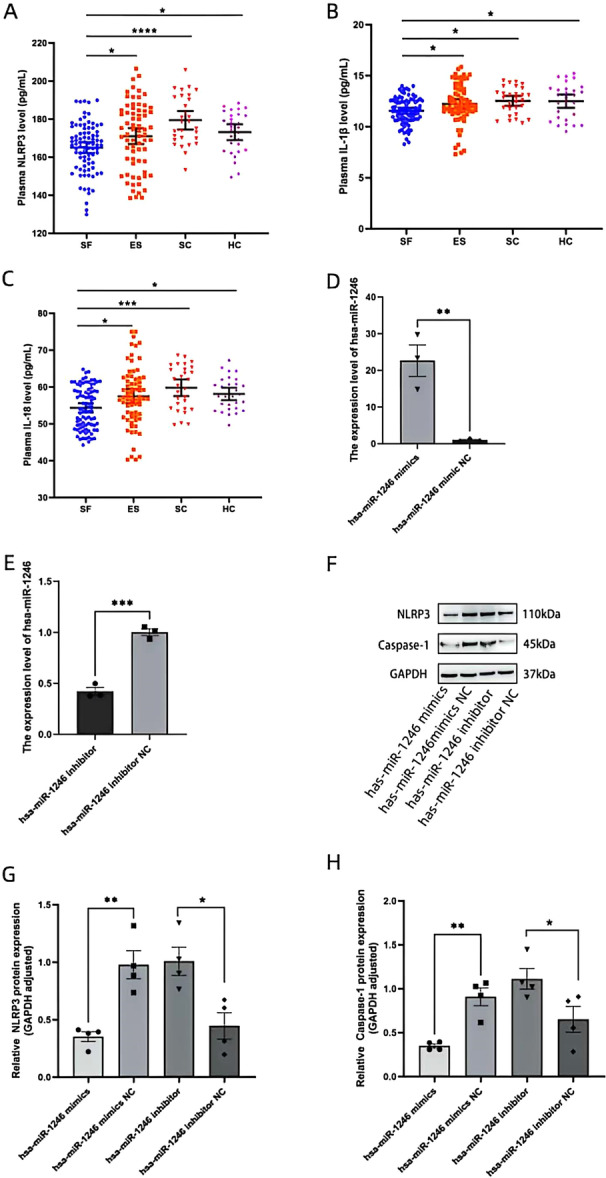
Exosome miR‐1246 negatively regulates the NLRP3 inflammasome expression. (A‐C) ELISA detected the expression levels of NLRP3, IL‐1β, and IL‐18 in plasma of different groups. (D‐E) RT‐qPCR verified cell transfection efficiency, *p < 0.05. (F‐H) Western Blot to analyze the effect of miR‐1246 on the expression of NLRP3 and Caspase‐1 protein.

ELISA assays revealed that the levels of NLRP3, IL‐1β, and IL‐18 in the SF group were significantly lower than those in the ES, SC, and HC groups (*p* < 0.01) (Figure 6A‐C). Based on our previous findings exhibiting a significant upregulation of miR‐1246 in patients with SF, we hypothesized that miR‐1246 may play a critical role in the pathogenesis of SF by negatively regulating the NLRP3 inflammasome. To validate this hypothesis, we successfully enhanced and inhibited miR‐1246 expression via cell transfection (Figure 6D‐E). Subsequent Western blotting revealed that the miR‐1246 mimic significantly decreased the protein levels of NLRP3 and caspase‐1, whereas the inhibitor significantly enhanced their expression (Figure 6F‐H).

### 3.7 The Diagnostic Value of Plasma Exosomal miR‐1246

**Figure 7:**
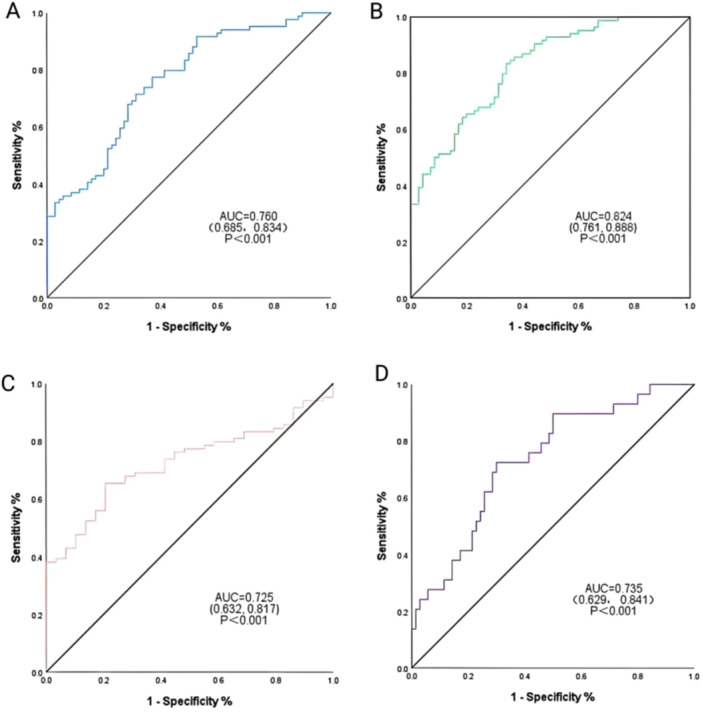
ROC curve analysis of plasma exosome miR‐1246. (A) ROC used to differentiate between SF and ES groups, *p* < 0.001. (B) miR‐1246 combined with RPR was used to differentiate ROC between SF and ES groups, *p* < 0.001. (C) ROC used to differentiate between SF and HC groups, *p* < 0.001.(D) ROC used to differentiate between SC and ES groups, *p* < 0.001. ROC. subject operating characteristics; AUC, area under the curve; ES, early syphilis; HC, healthy control; SC, syphilis cured; SF, serum fixation.

We generated ROC curves to assess the diagnostic value of plasma exosomal miR‐1246 in SF (Table 2 and Figure 7). ROC curve analysis revealed a significant difference in the expression of miR‐1246 between the SF and ES groups, with an AUC of 0.760, a sensitivity of 77.4%, and a specificity of 62.9% (*p* < 0.001, Figure 7A). After combining miR‐1246 with RPR, the AUC increased to 0.824, with a sensitivity of 83.3% and a specificity of 65.7% (*p* < 0.001, Figure 7B). The AUC was 0.725, sensitivity was 65.5%, and specificity was 79.3% when comparing the SF and HC groups (*p* < 0.001, Figure 7C). Furthermore, when comparing the SC and ES groups, the AUC was 0.735, sensitivity was 72.4%, and specificity was 70.0% (*p* < 0.001, Figure 7D).

**Table 2:** Diagnostic efficacy of plasma exo‐miR‐1246 in the differential diagnosis of SF.

Group	AUC (95%CI)	Sensibility%	Specificity %	Youden index	cutoff value	*p*
**SF vs ES**						
miR‐1246	0.760 (0.685,0.834)	77.4	62.9	0.403	0.538	< 0.001
miR‐1246 + RPR titer	0.824 (0.761,0.888)	83.3	65.7	0.490	0.503	< 0.001
**SF vs HC**	0.725 (0.632,0.817)	65.5	79.3	0.448	0.764	< 0.001
**SC vs ES**	0.735 (0.629,0.841)	72.4	70.0	0.424	0.656	< 0.001

## 4 Discussion

Syphilis has become a global public health problem due to its complex clinical manifestations and prolonged disease course [38]. Serum fixation (SF) is a unique clinical feature in which treponemal and non‐treponemal antibodies persist at low titers, making it significantly challenging to distinguish between active infection and SF. Several studies have focused on potential diagnostic markers for syphilis, including proteins, inflammatory markers, and outer membrane proteins [39, 40, 41]. However, most studies are still at the preliminary stage and their findings have not been widely translated into clinical practice. Exosomal miRNAs have attracted considerable attention due to their stability and easy application [18]. Studies have shown that miRNAs play a critical role in the pathogenesis of syphilis. For example, it was reported that miRNA‐146a, miRNA‐590, miRNA‐570, and miR‐197 are abnormally expressed in patients with syphilis and are involved in Treponema pallidum infection and the regulation of host immune responses [23, 24, 25].

miR‐1246 has been shown to play immunomodulatory roles in various diseases, including cancer, infectious diseases, and chronic inflammation [26, 27, 28, 29]. For instance, in the context of cancer, it has been observed to establish an immunosuppressive microenvironment [26]. Similarly, in rheumatoid arthritis and periodontitis, its levels have been shown to correlate positively with disease activity and inflammatory cytokine levels [27, 28]. Additionally, in viral infections, such as HIV‐1, it has been found to be packaged into exosomes, suggesting its potential role in chronic immune activation [29]. In infectious diseases, miR‐1246 is closely associated with infection with various pathogens. For example, Xu et al. [42] found that miR‐1246 is upregulated in HEV71‐infected human neuroblastoma cells, where it suppresses DLG3 and regulates cell signaling pathways. In addition, Gupta et al. [43] and Rego et al. [44] demonstrated that miR‐1246 can serve as a molecular indicator of the host response in malaria and Chagas disease, suggesting its potential application in disease diagnosis and prognosis. Our study also found that miR‐1246 is highly expressed in the plasma exosomes of patients with syphilis SF, indicating its important role in the development of syphilis SF. The function of miR‐1246 as an inflammatory regulator in multiple diseases is highly consistent with observations in the SF state of syphilis. Consequently, elevated expression levels of exosomal miR‐1246 during the SF stage may act as an inflammatory regulator, exerting an inhibitory effect on host‐related inflammatory hubs.

The NLRP3 inflammasome, a critical component of the host immune defense, has been implicated in several infectious and autoimmune diseases [30, 31, 32]. In normal conditions, the NLRP3 inflammasome exists in an inactive precursor form. When activated by tissue damage or pathogenic microorganisms, NLRP3 triggers the conversion of pro‐caspase‐1 to active caspase‐1, thereby promoting the production of inflammatory cytokines, such as IL‐1β and IL‐18 [45]. Studies have shown that the NLRP3 inflammasome plays a key role in the clearance of Treponema pallidum [33, 34]. Research has also demonstrated that low expression of the NLRP3 inflammasome is closely associated with syphilis serum fixation [35]. These findings suggest that inhibition of the NLRP3 inflammasome may contribute to the immune evasion of Treponema pallidum and the development of SF. In our study, we observed that both the NLRP3 inflammasome and inflammatory factors, such as IL‐1β and IL‐18, were significantly downregulated in the plasma of patients with SF. Furthermore, in vitro experiments confirmed that miR‐1246 negatively regulates the NLRP3 inflammasome. We speculated that miR‐1246 reduces the host's ability to clear *Treponema pallidum* by inhibiting the NLRP3 inflammasome, leading to the development of syphilis SF.

In this study, using GO and KEGG enrichment analysis, we elucidated numerous biological processes and pathways associated with the target genes of DEmiRNAs in patients with syphilis. These findings not only elucidate the potential role of miRNAs in the pathogenesis of syphilis but also provide potential targets and directions for future functional experiments and pharmacological studies through the analysis of these pathways. The ROC curve analysis confirmed the independent diagnostic value of plasma exosomal miR‐1246 in differentiating syphilis SF. Furthermore, it showed a synergistic advantage when combined with the rapid plasma reagin (RPR) test. Although miR‐1246 alone achieved moderate diagnostic accuracy (AUC = 0.760), its accuracy significantly improved after being combined with RPR testing (AUC = 0.824). These findings suggest that miR‐1246 can effectively compensate for the limited sensitivity and specificity of traditional serologic tests, offering significant potential for distinguishing between active infection and serum fixation.

This study collected independent samples from 212 patients with syphilis across two medical centers and validated miR‐1246 in plasma exosomes as a potential biomarker for syphilis seroconversion. Furthermore, we revealed that miR‐1246 may participate in syphilis seroconversion by negatively regulating NLRP3. This finding supports the potential of miR‐1246 as a biomarker for syphilis seroconversion.

### 4.1 Limitations

Despite interesting findings, this study was not without limitations. Firstly, the sample size was restricted to two hospitals, which may limit the generalizability of the results. Secondly, while this study confirmed the negative regulatory role of miR‐1246 on NLRP3 inflammasome activity, further validation is needed to confirm the direct molecular interactions between microRNA‐1246 and NLRP3. Thirdly, the absence of long‐term follow‐up data prevents a direct assessment of the association between the elevated levels of miR‐1246 and clinical outcomes. Moreover, the current research framework is characterized by a paucity of data from animal experiments.

### 4.2 Recommendations and Future Perspectives

From a translational medicine perspective, exploring intervention strategies targeting the miR‐1246‐NLRP3 axis may hold potential value. Research indicates that small‐molecule drugs can modulate the function of microRNAs [46, 47], proteasome‐targeting strategies can enhance host defense mechanisms [48], RIBOTAC aptamer‐based approaches can induce targeted degradation of microRNAs [49], and antisense oligonucleotide‐based microRNA inhibitors have advanced into clinical development and trial design phases [50]. Collectively, these interventional approaches (e.g., proteasome modulation, antisense/anti‐miRNA strategies) may offer complementary pathways for modulating the miR‐1246‐NLRP3 inflammasome axis to verify whether they can improve the serological fixation of syphilis. It is important to note that these research directions are proposed solely as potential avenues for future mechanistic and translational studies and do not represent conclusions of this study.

## 5 Conclusion

Our findings suggest that the levels of miR‐1246 in plasma exosomes are significantly elevated in patients with syphilis SF. The potential mechanism underlying this phenomenon may involve negative regulation of NLRP3 inflammasome activity, thereby contributing to SF formation. Furthermore, the study indicated that miR‐1246 has the potential to serve as a biological marker for SF. However, this finding necessitates further validation through the analysis of larger clinical datasets.

## Author Contributions

**Yue Mou:** writing – manuscript (lead), validation, data management. **Caifeng He:** resources (equal), funding acquisition. **Wenhao Cheng:** methodology (equal), funding acquisition. **Fanxiang Wang:** fieldwork. **Chaochao Ji:** methodology (Equal); **Xinting Wang:** fieldwork. **Hong Ren:** writing – review and editing, conceptualization. **Wenlong Hu:** writing – review and editing, methodology (lead), supervision, funding acquisition.

## Conflicts of Interest

The authors declare no conflicts of interest.

## Supporting information


Supporting File:

## Data Availability

The data sets generated during and/or analysed during the current study are available from the corresponding author on reasonable request.
