# Supplementary material for: Exosomal miR‐1246 in Syphilis Serofast State: Diagnostic Value and NLRP3 Inflammasome Suppression
Source: Immun Inflamm Dis. 2026 Apr 9;14(4):e70434. doi: 10.1002/iid3.70434 (PMC13066714; doi:10.1002/iid3.70434)
Supplement: Supplementary file 1 — Supporting File: [file IID3-14-e70434-s001.docx]

**Supplementary Tables**

**Table S1** Primer sequences used for reverse transcription and PCR.

| Sequence name | Primer sequences(5'-3') |
| --- | --- |
| cel-miR-39 mimics | UCACCGGGUGUAAAUCAGCUUG |
| cel-miR-39-3p RT primer | GTCGTATCCAGTGCAGGGTCCGAGGTATTCGCACTGGATACGACCAAGCT |
| cel-miR-39-3p forward primer | CGGCGTCACCGGGTGTAAATC |
| cel-miR-39-3p reverse primer | CAGTGCGTGTCGTGGAGTC |
| has-miR-1246 RT primer | GTCGTATCCAGTGCAGGGTCCGAGGTATTCGCACTGGATACGACCCTGCT |
| has-miR-1246 forward primer | GCGCGAATGGATTTTTGG |
| has-miR-1246 reverse primer | AGTGCAGGGTCCGAGGTATT |
| U6 RT primer | GTCGTATCCAGTGCAGGGTCCGAGGTATTCGCACTGGATACGACAAAATA |
| U6 forward primer | AGAGAAGATTAGCATGGCCCCTG |
| U6 reverse primer | ATCCAGTGCAGGGTCCGAGG |

**Table S2** The microRNA mimics and inhibitor sequences used for cell transfection.

| Name | Primer sequences(5'-3') |
| --- | --- |
| miR-1246 mimics | AAUGGAUUUUUGGAGCAGG |
| miR-1246 inhibitor | CCUGCUCCAAAAAUCCAUU |
